# From Elective to Emergent: The Clinical Consequences of Delayed Umbilical Hernia Repair

**DOI:** 10.7759/cureus.106322

**Published:** 2026-04-02

**Authors:** Oscar Castillo, Srilekha Ravella, Sohrab Mardani, Soshiant Raeesian, Khaled Saed, Joshua Simon

**Affiliations:** 1 Medicine, Burrell College of Osteopathic Medicine, Las Cruces, USA; 2 Surgery, Larkin Community Hospital, Miami, USA; 3 General Surgery, Delray Medical Center, Delray Beach, USA

**Keywords:** abdominal wall defect, emergent repair, incarcerated hernia, robotic-assisted surgery, umbilical hernia

## Abstract

Umbilical hernias are commonly diagnosed ventral abdominal wall defects and are frequently managed electively. Diagnosis is primarily clinical but may be supported by imaging modalities such as computed tomography (CT), ultrasound (US), or magnetic resonance imaging (MRI). Although most patients remain asymptomatic, surgical intervention is required for definitive repair. However, delayed repair can transform a low-risk outpatient procedure into a surgical emergency associated with increased morbidity and limited operative options, including the inability to place mesh. The absence of mesh reinforcement is associated with substantially higher recurrence rates, even in small hernias. Therefore, patient education and elective surgical repair of umbilical hernias should occur before complications arise that restrict mesh use.

We present the case of a 64-year-old man with a previously diagnosed umbilical hernia who deferred elective repair and subsequently presented with acute abdominal pain due to incarceration. Emergent robotic-assisted repair was performed, requiring extensive lysis of adhesions and primary fascial closure with suture. Intraoperative findings of bowel edema and serosanguineous fluid rendered the surgical field contaminated, ultimately precluding mesh placement. This case highlights the tangible cost of delayed elective umbilical hernia repair. Deferral of intervention can lead to emergent presentation, limit surgical options, and increase the risk of recurrence. The true cost of delay is not the emergent operation itself, but the permanent loss of the ability to perform the most durable repair possible.

## Introduction

Among adults, umbilical hernias represent a notable proportion of abdominal wall hernias and are most often acquired defects caused by progressive fascial weakening [1]. An umbilical hernia represents a defect in the abdominal wall fascia, allowing intra-abdominal contents to protrude through a weakened area. Incarceration refers to the trapping of hernia contents, while strangulation describes compromised blood supply to the entrapped tissue, which can lead to ischemia. They account for approximately 6-14% of all abdominal wall hernias and are second in frequency only to inguinal hernias [1,2]. Progressive enlargement of the fascial defect carries an increasing risk of incarceration or strangulation [3], although larger defects may allow freer movement of hernia contents and therefore may have a comparatively lower risk of incarceration.

Long-term cohort data demonstrate that even small fascial defects repaired without mesh carry recurrence rates exceeding 20%, highlighting the importance of durable elective repair [4]. Mesh-based repair, however, has been associated with significantly lower recurrence rates compared with primary suture repair, with studies demonstrating up to a 50% reduction in recurrence risk [4,5].

Elective repair, particularly with mesh reinforcement, is associated with more durable outcomes and reduced tension on fascial closure, contributing to improved long-term results [4,5]. By comparison, emergent repair frequently occurs in contaminated or inflamed surgical fields, where mesh placement may be contraindicated due to increased infection risk, thereby necessitating primary closure and increasing recurrence risk [6]. This report describes a patient with significant comorbidities who deferred elective umbilical hernia repair and ultimately required emergent robotic intervention, during which contamination precluded safe mesh placement based on intraoperative assessment. This case demonstrates the importance of early elective management to optimize operative conditions and improve long-term outcomes.

## Case presentation

A 64-year-old man with a two-year history of a previously reducible umbilical hernia presented with acute periumbilical pain, nausea, and a newly irreducible umbilical mass. He reported no bowel movement or flatus for 24 hours.

His medical history was significant for obesity (body mass index (BMI) 37 kg/m²), cardiopulmonary comorbidities including diastolic heart failure and pulmonary hypertension, and prior abdominal surgery (appendectomy), consistent with reduced cardiopulmonary reserve.

On initial evaluation, vital signs were measured as a blood pressure of 182/114 mmHg, indicating severe hypertension, and a heart rate of 105 beats per minute, demonstrating mild tachycardia, likely reflecting an acute physiologic stress response in the setting of acute abdominal pathology.

Physical examination revealed a 5 cm tender, non-reducible umbilical bulge without skin discoloration. Given concern for incarceration and possible bowel obstruction, laboratory studies were obtained and demonstrated mild leukocytosis and a normal lactate level (Table 1), suggesting an inflammatory process without evidence of significant tissue hypoperfusion. Hemoglobin, creatinine, and electrolyte levels were within normal limits, indicating no evidence of anemia, renal dysfunction, or significant metabolic dysfunction.

**Table 1:** Laboratory findings at the time of initial presentation Initial laboratory studies obtained during emergency department evaluation are summarized. Laboratory results on presentation demonstrate mild leukocytosis (white blood cell count 12.2×10⁹/L). Hemoglobin (14.8 g/dL) and platelet count (215×10³/µL) are within normal limits. Electrolytes are unremarkable, with potassium at the lower limit of normal (3.5 mEq/L). Renal function is preserved (creatinine 1.2 mg/dL), and lactate is normal (0.9 mmol/L), indicating no biochemical evidence of hypoperfusion.

Laboratory test	Patient value	Reference range
White blood cell count	12.2×10⁹/L	4.0-11.0×10⁹/L
Hemoglobin	14.8 g/dL	13.5-17.5 g/dL (male)
Platelet count	215×10^3^/µL	150-400×10^3^/µL
Sodium	142 mEq/L	135-145 mEq/L
Potassium	3.5 mEq/L	3.5-5.0 mEq/L
Creatinine	1.2 mg/dL	0.6-1.3 mg/dL
Lactate	0.9 mmol/L	0.5-1.0 mmol/L

**Figure 1:**
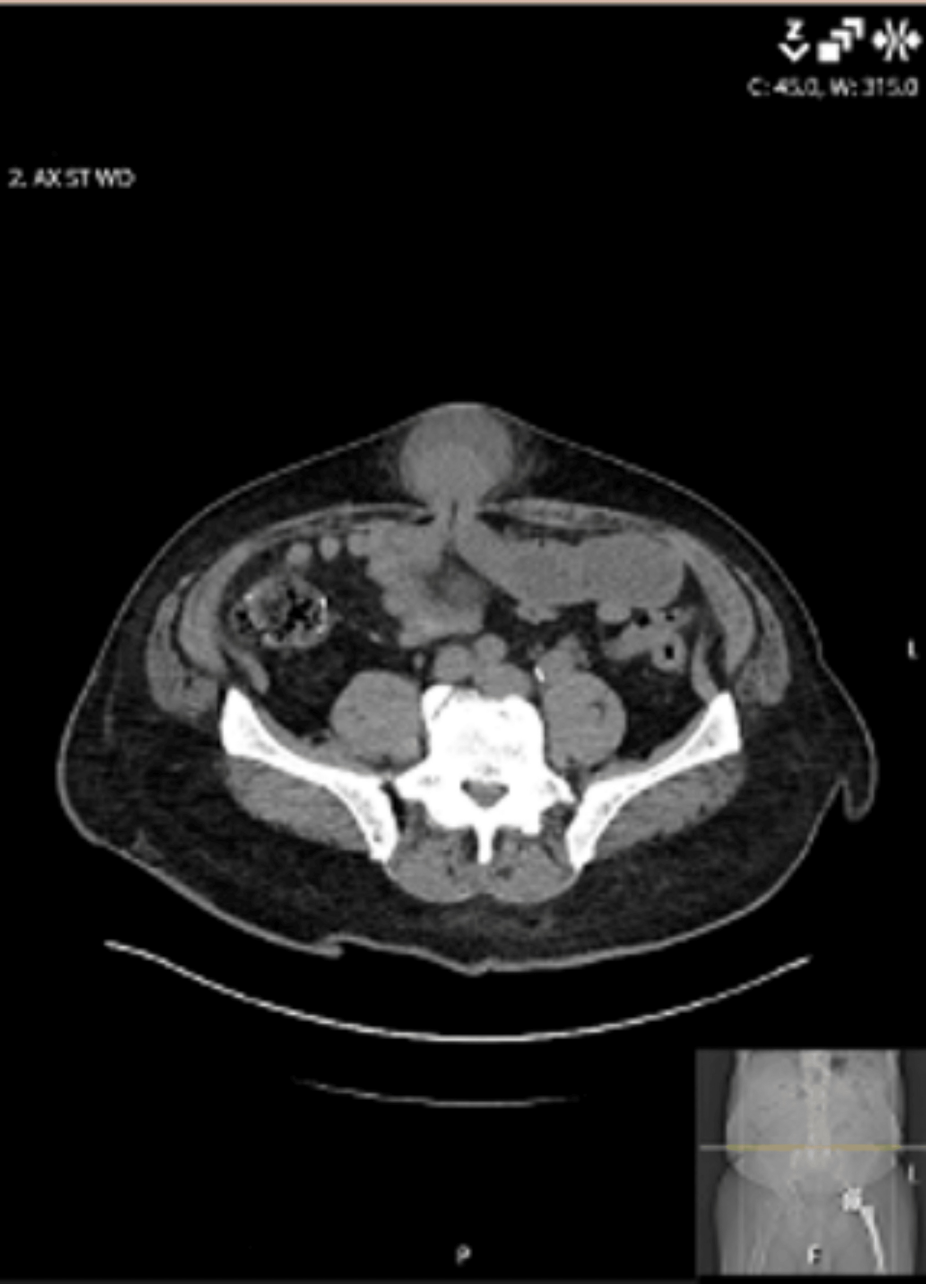
Axial computed tomography image demonstrating an umbilical hernia containing the small bowel and omentum

**Figure 2:**
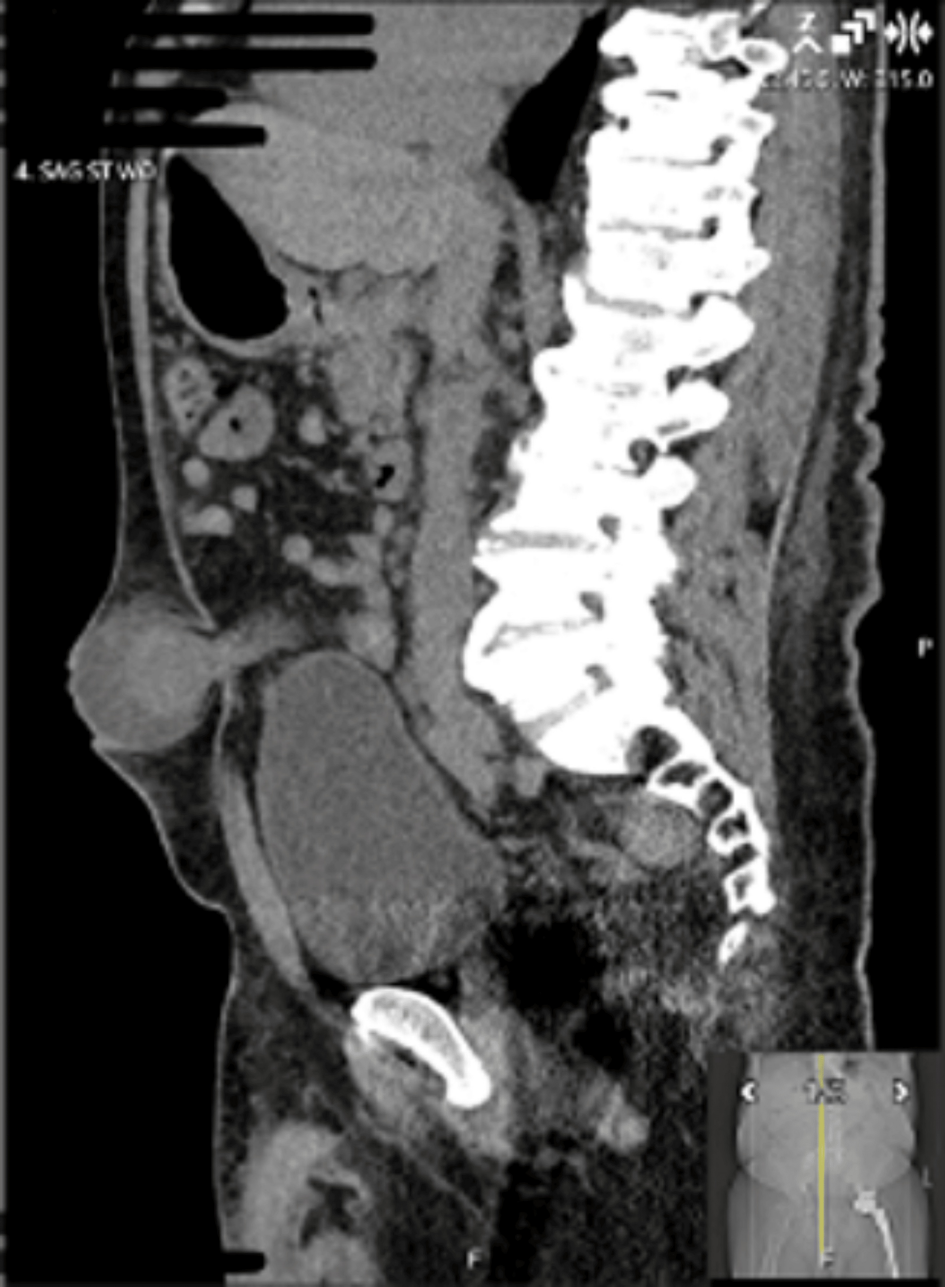
Sagittal computed tomography image demonstrating a protruding umbilical hernia sac consistent with incarceration

Although an umbilical hernia is primarily a clinical diagnosis, computed tomography (CT) of the abdomen and pelvis with intravenous contrast was obtained in this case to evaluate for complications, including incarceration, bowel obstruction, and ischemia, as well as to assist in operative planning by assessing bowel viability and the extent of hernia contents. Imaging demonstrated an incarcerated umbilical hernia containing small bowel and omentum, consistent with closed-loop obstruction, without definitive radiographic signs of bowel ischemia such as pneumatosis or portal venous gas (Figures 1-2).

Given these findings, the patient was taken urgently to the operating room for robotic-assisted repair. A robotic-assisted approach was selected to allow enhanced visualization, improved dexterity, and precise adhesiolysis in the setting of suspected dense intra-abdominal adhesions. Intraoperatively, upon insufflation, dense intra-abdominal adhesions involving the omentum and small bowel adjacent to the hernia sac were observed. A 4×5 cm fascial defect contained edematous but viable bowel.

During reduction, serosanguineous fluid was encountered, raising concern for early ischemic change and a potentially contaminated operative field. Mesh placement was therefore deferred based on intraoperative findings and surgeon judgment regarding infection risk. No intraoperative photograph was obtained; however, the operative findings described correlate with the preoperative CT imaging (Figures 1-2). 

Robotic lysis of adhesions was completed, the incarcerated bowel reduced, and the fascial defect closed primarily with a running barbed suture under reduced pneumoperitoneum. Estimated blood loss was 5 mL, and no intraoperative complications occurred.

The patient tolerated the procedure well, resumed oral intake on postoperative day 2, and was discharged home on postoperative day 3. At the initial outpatient follow-up, he was noted to be recovering appropriately without complications.

At approximately five months postoperatively, the patient was clinically evaluated, and physical examination documented a soft, non-tender, and non-distended abdomen without reported abdominal pain or gastrointestinal symptoms. No evidence of hernia recurrence or wound-related complications was identified at that time.

## Discussion

This case illustrates how delayed elective repair can transform a low-risk outpatient procedure into a complex emergent operation. The patient's comorbidities, including obesity, cardiopulmonary disease, and prior orthopedic surgery, predisposed him to hernia formation through chronic increases in intra-abdominal pressure and impaired tissue healing [7]. Obesity, in particular, is an established risk factor for both hernia recurrence and surgical site complications [8]. Delays in operative management of emergent hernias have been associated with increased morbidity, further emphasizing the importance of timely surgical intervention [9].

Umbilical hernias are often perceived by patients as benign due to their slow growth and intermittent symptoms [10]. However, longitudinal data demonstrate a measurable risk of incarceration among patients managed non-operatively, with a cumulative incidence of 1.24% at one year and 2.59% at five years [11]. Thus, "watchful waiting" for umbilical hernias is not risk-neutral but represents a decision that trades short-term convenience for the potential of a significantly more morbid emergent presentation. 

During elective repair, mesh reinforcement in a clean surgical field provides tensile strength, reduces tension on the fascial closure, and significantly decreases recurrence [12]. Mesh integration into host tissue depends on fibrovascular ingrowth into well-perfused tissue. However, the presence of bowel edema and serosanguineous fluid, as observed in this case, disrupts this process, increasing the risk of bacterial colonization, biofilm formation, and impaired wound healing [13]. Long-term outcomes data suggest that the benefits of mesh reinforcement may be partially offset by mesh-related complications, supporting selective avoidance of mesh implantation in contaminated fields based on intraoperative assessment and surgeon judgment [14].

Contaminated fields convert what would normally be a prosthetic reinforcement procedure into a suture-only repair, known to carry significantly higher recurrence rates [15]. Primary suture repair of umbilical hernias is associated with higher recurrence rates compared with mesh reinforcement, with meta-analytic data demonstrating a 52% lower risk of recurrence with mesh repair [16]. Therefore, the inability to place mesh during emergent repair does not merely change operative technique as it fundamentally alters the long-term prognosis of the repair. However, primary suture repair remains a viable option, and mesh reinforcement may be considered in the setting of recurrence.

Primary care providers play a critical role in early referral and patient education, as hernias are often first identified in outpatient settings. Early surgical consultation allows for elective planning under optimal conditions before contamination, incarceration, or tissue compromise occurs. This case demonstrates that the true cost of delayed repair is not financial, but clinical, specifically, the permanent loss of the ability to perform the most durable repair possible.

## Conclusions

This case highlights the pivotal role of timely elective repair in umbilical hernia management. While robotic-assisted repair allowed a safe and efficient emergent operation, the contaminated field prevented mesh placement, inherently increasing recurrence risk. Had this patient undergone elective repair prior to incarceration, mesh reinforcement could have been performed under optimal conditions, ensuring a durable outcome with minimal morbidity. Surgeons and primary care providers should educate patients on the importance of early elective intervention to prevent avoidable emergencies and the clinical consequences of delayed repair, including the loss of optimal surgical options.
